# Unusual Cutaneous Lesions in a 53-Year-Old Female: A Potential Indicator of Underlying Renal Cell Carcinoma

**DOI:** 10.7759/cureus.48783

**Published:** 2023-11-14

**Authors:** Samuel E Audet, Elena Koleva, Hayder N Alhameedi, Samuel Ashcroft, Ayesha Imran

**Affiliations:** 1 Dermatology, Mid and South Essex NHS Foundation Trust, Basildon, GBR; 2 Dermatology, Mid and South Essex NHS Foundation Trust, Southend, GBR; 3 Internal Medicine, Mid and South Essex NHS Foundation Trust, Southend, GBR

**Keywords:** fumarate hydratase, genetic skin disease, genetic renal diseases, hereditary leiomyoma and renal cell carcinoma, renal cell carcinoma (rcc)

## Abstract

Hereditary leiomyomatosis and renal cell cancer (HLRCC) is a rare autosomal dominant genetic disorder resulting from mutations in the fumarate hydratase (FH) gene. It is characterised by a predisposition to cutaneous and uterine leiomyomas (fibroids) and an aggressive form of renal cell carcinoma (RCC). We report the case of a 53-year-old female who presented with an unusual rash in the context of a personal and family history of uterine leiomyomas requiring hysterectomy. A skin biopsy confirmed cutaneous leiomyomas and subsequent genetic testing demonstrated a pathogenic heterozygous mutation on exon 7 of the FH gene, confirming a diagnosis of HLRCC. Due to the recognised association with renal cell carcinoma in this syndrome, abdominal imaging was performed, which excluded RCC, and the patient was commenced on lifelong surveillance with annual abdominal magnetic resonance imaging.

## Introduction

Hereditary leiomyomatosis and renal cell cancer (HLRCC) is a rare autosomal dominant disorder caused by germline mutations in the fumarate hydratase (FH) gene, which encodes the FH enzyme, a key component of the tricarboxylic acid (TCA) cycle [1]. Patients are predisposed to develop cutaneous leiomyomas, uterine leiomyomas (fibroids) in women, and early-onset aggressive renal cell carcinomas [2]. Due to its rarity and the often insidious nature of its clinical presentation, HLRCC remains a diagnostic challenge for clinicians. Early identification of the syndrome with appropriate subsequent screening and genetic counselling is crucial for improving the outcomes for patients and families at risk of HLRCC [3].

## Case presentation

A 53-year-old female was referred to our secondary care general outpatient dermatology clinic with a 10-year history of lesions affecting her left hypochondrium that had progressively increased in number and were associated with worsening intermittent pain and pruritis, prompting her to seek medical evaluation. She had a history of mild scalp and flexural psoriasis and had undergone a hysterectomy for symptomatic uterine fibroids at 38 years of age. She took no regular medications, and no other pertinent medical history was reported. Her family medical history revealed that both her mother and sister had undergone hysterectomies in their forties due to symptomatic uterine fibroids. There was no family history of dermatological disease.

**Figure 1:**
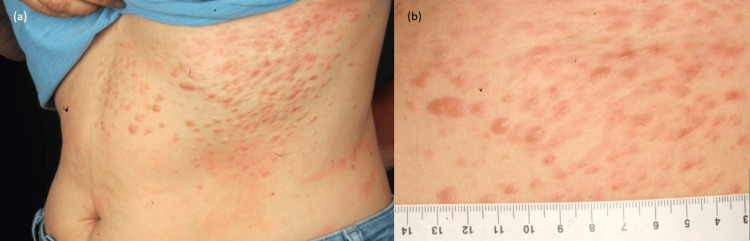
(a) Cutaneous lesions over the left abdomen in a zosteriform pattern. (b) Close-up image showing multiple red-to pinkish papules and nodules with some areas of confluence.

Dermatological examination revealed multiple firm, tender papulonodular flesh-coloured lesions over the left hypochondrium and epigastrium in dermatomal distribution (Figure 1).

Blood tests including full blood count, renal function, electrolytes, liver function, lipid profile, thyroid function, and coagulation were unremarkable. Given the unusual appearance of the skin lesions, she underwent a diagnostic biopsy, which demonstrated spindle-shaped smooth muscle cells arranged in fascicles, without evidence of atypia, suggestive of cutaneous leiomyoma. The diagnosis of HLRCC was suspected based on the personal and family history of early onset uterine leiomyomas. Given the association of HLRCC with renal cell carcinoma, a computerised tomography scan of the abdomen and pelvis was performed, showing no renal abnormalities. The patient underwent genetic testing, which revealed a pathogenic heterozygous mutation on exon 7 of the FH gene, confirming the diagnosis of HLRCC. After consulting with a specialist in rare dermatological conditions, it was recommended that the patient should have annual monitoring for cutaneous leiomyosarcoma and annual monitoring for renal cell carcinoma with magnetic resonance imaging.

## Discussion

There is variable nomenclature of the condition in the literature, which was first described by Reed in 1973 as a genetic disorder characterised by multiple cutaneous and uterine leiomyomas. It was subsequently termed multiple cutaneous and uterine leiomyomas (MCUL), or ‘Reed’s Syndrome’. The term HLRCC became more widely used after recognition of the association between the cutaneous and uterine leiomyomas and renal cell carcinoma in the early 2000s; however, all these terms describe the same disorder.

Although the pathogenesis is incompletely understood, loss of function of the FH enzyme, which is responsible for catalysing the conversion of fumarate to malate in the Krebs cycle, plays a central role. Mutations in this enzyme can result in the accumulation of intracellular fumarate, triggering a pseudohypoxic cellular state [1,4]. Mitochondrial FH loss may lead to fumarate accumulation, stabilising transcription factors such as HIF 1α and 2α, which in turn activate proliferative genes that create a conducive environment for tumour growth [1].

The genetic mutation has been identified in more than 300 families worldwide [5]. Cutaneous leiomyomas are often the first noticeable symptom in HLRCC, occurring in over three-quarters of patients with the syndrome [6], and therefore first presentation is often to a dermatologist. The age of onset is typically in the second and third decades of life but may present later. They present as painful, pink-to-reddish-brown papules or nodules, usually distributed over the trunk and limbs, approximately 0.5-2.5 cm in size and can be grouped, dermatomal or linear in distribution [7]. Histological features of these lesions are typical for leiomyomas with interlacing bundles of elongated smooth muscle cells with eosinophilic cytoplasm.

Uterine leiomyomas (fibroids) will develop in the vast majority of women with HLRCC, with many affected women requiring surgical intervention due to the size and number of fibroids, and associated symptoms. In cases where women with HLRCC present with both cutaneous and uterine leiomyomas, almost half will require a hysterectomy at or below the age of 30 [8].

The most significant concern in patients with HLRCC is the increased risk of renal cell carcinoma (RCC), particularly type II papillary RCC, which can manifest at a younger age and with a higher likelihood of metastases. Around 12% of patients with HLRCC have detectable RCC at the time of diagnosis, and it is estimated that the lifetime risk of developing RCC in patients with HLRCC is 21% [9]. Approximately half of the cases of type II papillary RCC are metastatic at the time of diagnosis in patients with HLRCC [10]. Although type II papillary RCC is the most common histological subtype seen in HLRCC, other subtypes of RCC such as collecting duct, tubulopapillary, solid and mixed histological types have also been reported [11].

Detecting RCC in patients with HLRCC poses a diagnostic challenge, as although RCC can present in some patients with symptoms such as haematuria, loin pain and a palpable abdominal mass, most patients with type II papillary RCC are asymptomatic at the time of diagnosis [12]. Surveillance imaging is therefore crucial for patients with a confirmed diagnosis of HLRCC. Although there is no definitive guideline on surveillance, some literature suggests contrast-enhanced magnetic resonance imaging as the modality of choice, with 1 to 3 mm slices through the kidneys on an annual basis [6]. Where RCC is identified, prompt surgical intervention with wide surgical margins and consideration of retroperitoneal lymph node dissection is recommended even for small tumours, given the aggressive metastatic potential of HLRCC-associated RCC [2].

FH deficiency, which results from the autosomal recessive inheritance of two pathogenic variants in the FH gene, has also been documented in the medical literature. Clinically this manifests in the neonatal period with seizures, encephalopathy, and failure to thrive [13]. Autosomal recessive FH deficiency is extremely rare, with approximately 100 cases reported worldwide, and compared to HLRCC, it is associated with a much worse prognosis, with most affected individuals not surviving infancy [14].

## Conclusions

Diagnosis of HLRCC requires an index of suspicion in suspected cases, along with an in-depth medical and family history, and thorough examination to identify early-onset uterine fibroids, cutaneous leiomyomas, and renal cell carcinoma. Diagnostic procedures should include a skin biopsy for confirmation of cutaneous leiomyomas, and abdominal and pelvic ultrasound to evaluate uterine fibroids. Genetic testing is required for a definitive diagnosis. A multidisciplinary team approach is crucial to providing holistic care to patients with suspected or confirmed HLRCC. The cornerstone of HLRCC management is vigilant RCC screening and ongoing surveillance, typically with annual contrast-enhanced magnetic resonance imaging of the kidneys. Effective pain management for multiple cutaneous leiomyomas, and long-term monitoring for leiomyosarcoma, alongside regular gynaecological assessments for uterine fibroids, forms an integral part of the holistic care strategy. Furthermore, genetic counselling is extremely important, particularly for individuals with a family history of HLRCC, to detect asymptomatic at-risk relatives early, and to begin appropriate RCC surveillance. The collaborative effort of the multidisciplinary team, encompassing geneticists, nephrologists, dermatologists, gynaecologists, and oncologists, ensures a coordinated and patient-centred management pathway, ultimately improving outcomes for individuals with HLRCC.
